# Neuropharmacological insight into preventive intervention in posttraumatic epilepsy based on regulating glutamate homeostasis

**DOI:** 10.1111/cns.14294

**Published:** 2023-06-12

**Authors:** Yuan Gao, Ning Liu, Juan Chen, Ping Zheng, Jianguo Niu, Shengsong Tang, Xiaodong Peng, Jing Wu, Jianqiang Yu, Lin Ma

**Affiliations:** ^1^ Department of Pharmacology Ningxia Medical University Yinchuan China; ^2^ Hunan Province Key Laboratory for Antibody‐Based Drug and Intelligent Delivery System, School of Pharmaceutical Sciences Hunan University of Medicine Huaihua China; ^3^ Ningxia Key Laboratory of Craniocerebral Diseases of Ningxia Hui Autonomous Region Ningxia Medical University Yinchuan China

**Keywords:** excitatory neurotoxicity, glutamate homeostasis, neuropharmacological research, posttraumatic epilepsy, preventive treatment, traumatic brain injury

## Abstract

**Background:**

Posttraumatic epilepsy (PTE) is one of the most critical complications of traumatic brain injury (TBI), significantly increasing TBI patients' neuropsychiatric symptoms and mortality. The abnormal accumulation of glutamate caused by TBI and its secondary excitotoxicity are essential reasons for neural network reorganization and functional neural plasticity changes, contributing to the occurrence and development of PTE. Restoring glutamate balance in the early stage of TBI is expected to play a neuroprotective role and reduce the risk of PTE.

**Aims:**

To provide a neuropharmacological insight for drug development to prevent PTE based on regulating glutamate homeostasis.

**Methods:**

We discussed how TBI affects glutamate homeostasis and its relationship with PTE. Furthermore, we also summarized the research progress of molecular pathways for regulating glutamate homeostasis after TBI and pharmacological studies aim to prevent PTE by restoring glutamate balance.

**Results:**

TBI can lead to the accumulation of glutamate in the brain, which increases the risk of PTE. Targeting the molecular pathways affecting glutamate homeostasis helps restore normal glutamate levels and is neuroprotective.

**Discussion:**

Taking glutamate homeostasis regulation as a means for new drug development can avoid the side effects caused by direct inhibition of glutamate receptors, expecting to alleviate the diseases related to abnormal glutamate levels in the brain, such as PTE, Parkinson's disease, depression, and cognitive impairment.

**Conclusion:**

It is a promising strategy to regulate glutamate homeostasis through pharmacological methods after TBI, thereby decreasing nerve injury and preventing PTE.

## Graphical abstract

This paper discusses the effect of traumatic brain injury (TBI) on glutamate homeostasis and its role in the occurrence and development of posttraumatic epilepsy (PTE). The molecular pathway to restore glutamate homeostasis after TBI is explored for the first time, and the pharmacological research progress of PTE prevention by regulating glutamate homeostasis is summarized.

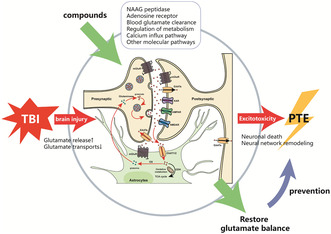

## 1 INTRODUCTION

Traumatic brain injury (TBI) is an alteration in brain function or other pathological injuries of brain tissue caused by external force.^1^ Each year, an estimated 69 million individuals worldwide suffer from TBI, most of which are mild (81%) and moderate (11%).^2^ Posttraumatic epilepsy (PTE) is recurrent epilepsy secondary to TBI and one of the most critical complications of TBI. Compared with the general population, patients with severe TBI have a 30‐fold increase in the incidence rate of epilepsy, and this risk is higher among soldiers exposed to missile and blast injuries.^3^ According to the time interval between TBI and PTE, posttraumatic seizures (PTS) are usually divided into immediate seizures (<24 h), early seizures (24 h–7 days), and late seizures (>7 days). The risk of PTE was significantly correlated with gender, early traumatic seizures (EPT), time of loss of consciousness (LOC), subdural hematoma (SDH), location of brain contusion, cranial surgery, and TBI severity.^4^

The current theory holds that the development of acquired epilepsy (AE), including PTE, mainly goes through three phases: acute brain injury, the latent period of epilepsy, and the spontaneous recurrent period of chronic epilepsy.^5^ After the acute brain injury phase, the brain will gradually change at the histological, cytological and molecular levels, contributing to recurrent epilepsy. With the discovery of risk factors and biomarkers of PTE, people can now better predict PTE. If we can take corresponding measures in the window between TBI and late recurrent epilepsy, we may be able to prevent the onset of PTE.^6^ Therefore, from the theoretical point of view, PTE is potentially preventable epilepsy.^7^

In current clinical practice, the prevention and treatment of PTE mainly include the following strategies: (1) after the occurrence of severe TBI, take preventive measures to prevent the occurrence of early PTS in time; (2) when late PTE occurs, take first‐line antiepileptic drugs (AEDs) that are effective for focal epilepsy; (3) for patients with drug‐resistant PTE or unable to tolerate drug side effects, surgical treatment can be selected.^8^ Unfortunately, the existing AEDs do not seem ideal for reducing the risk of late PTE. Phenytoin sodium and levetiracetam are the most commonly used AEDs to prevent PTE in clinical applications.^9^, ^10^ Still, previous studies have shown that early administration of AEDs can reduce the risk of early PTS compared with placebo or standard care. Still, there was no evidence that AEDs can reduce the risk of late seizures or mortality.^11^ Another retrospective study also showed that the occurrence of late PTS is not relieved by preventive treatment.^12^ Craniotomy and decompressive surgery showed the same situation.^13^ The complex and unclear pathogenesis may have contributed to the failure of traditional AEDs in preventing PTE.

The mechanism of PTE is complex, involving blood–brain barrier damage, inflammatory reaction, and excitotoxicity. The imbalance between excitatory and inhibitory neurotransmitter transmission and the consequent excitotoxicity is one of the fundamental causes of symptomatic epilepsy and the development and maintenance of PTE. Glutamate is the most abundant neurotransmitter in the nervous system, closely related to excitatory signal transmission between neurons and neuroplasticity of the normal brain.^14^ TBI causes increased release and transport abnormalities of glutamate in the brain, leading to the accumulation of glutamate in the neuronal extracellular space. Abnormally accumulated glutamate mediates excitotoxic effects via glutamate receptors on cells, leading to neuronal damage and even death. In the acute phase of TBI, glutamate‐induced neuronal injury and alterations in neural networks may increase the susceptibility to epilepsy and contribute to recurrent seizures. Elucidating the role of the glutamate system in the development of PTE and its intervention pathways will facilitate the screening and discovery of preventive drugs for PTE.

In this review, we will systematically expound on the effects of TBI on glutamate homeostasis and the related role of glutamate involvement in the onset and development of PTE. In addition, we will review the pharmacological research on the molecular pathways regulating glutamate homeostasis after TBI to provide a reference for drug research and development for the intervention of PTE.

## 2 ABNORMAL GLUTAMATE HOMEOSTASIS AND PTE

### 2.1 Glutamate‐glutamine cycle

The complete glutamate system involves glutamate synthesis, presynaptic glutamate release, the interaction between glutamate and glutamate receptors, and glutamate uptake by glutamate transporters. Glutamate is released from the presynapse to the synaptic cleft in a calcium‐dependent manner in response to neuronal depolarization. Glutamate released into the extracellular space can exert excitatory synaptic transmission by binding to several ionotropic glutamate receptors (iGluRs) and metabotropic glutamate receptors (mGluRs) subtypes at the postsynapse or spillover from the synaptic cleft. In contrast, ambient glutamate participates in physiological and pathological changes.^15^ Postsynaptic ionotropic receptors include N‐methyl‐D‐aspartate receptor (NMDAR), α‐Amino‐3‐hydroxy‐5‐methyl‐4‐isoxazole propionic acid receptor (AMPAR), and kainic acid receptor (KAR).^16^ Under normal physiological conditions, glutamate released from glutamatergic nerve terminals acts on postsynaptic receptors, causing depolarization and releasing Mg^2+^ cations from NMDA receptor channels, allowing Ca^2+^ to pass through pores. AMPAR is usually co‐expressed with NMDAR at the synapse. They participate in synaptic plasticity, involving learning, memory, excitotoxicity, and neuroprotection. Metabotropic glutamate receptors (mGluRs) are Class C 7‐transmembrane domain (7‐TMD) G‐protein coupled receptors (GPCRs). mGluR dimers bind glutamate, which triggers a conformational change of the receptor and the initiation of intracellular signaling, participating in the modulation of neurotransmission throughout the nervous system.^17^ According to G‐protein coupling, sequence homology, and ligand selectivity, mGluRs can be subdivided into three groups. Group I (mGlu1 and mGlu5) are coupled with Gq/11 and related signal pathways, group II (mGlu2 and mGlu3) and group III (mGlu4, 6, 7, 8) are coupled with Gi/o signaling.^18^ Compared with ligand‐gated ion channels (NMDA, AMPA, and KAR) responsible for rapid excitatory transmission, mGluRs have a greater regulatory effect, helping fine‐tune synaptic efficacy and control the accuracy and sharpness of transmission. Its allosteric regulator has various pharmacological effects on central system diseases.^19^

The extrasynaptic glutamate mainly originates from the spillover from synapses,^15^, ^20^ as well as astrocytic^21^ and microglial^22^ release. Glutamate released by neurons is usually not metabolized in the extracellular space. To maintain the normal concentration of glutamate in the extracellular space and avoid its excitotoxicity, excitatory amino acid transporters (EAATs) located on astrocytes and neurons remove glutamate by reuptake. EAATs are members of the solute carrier 1 (SLC1) family of transmembrane amino acid transporters that transport glutamate and aspartate across the plasma membrane. Five different EAATs have been identified, which are EAAT1‐5. The homologous transporters corresponding to rodents are named GLAST (EAAT1), GLT‐1 (EAAT2), EAAC1 (EAAT3), EAAT4 and EAAT5.^23^, ^24^ Glutamate transporter subtypes have a different distribution in neurons and astrocytes. EAAT1 exists in neurons and astrocytes and is most abundant in Bergman glial cells in the cerebellar molecular layer but also in the cortex, hippocampus, and deep cerebellar nucleus.^25^ The heterozygous mutation of EAAT1 can lead to a decrease in glutamate uptake, which causes neuronal hyperexcitability and induces seizures, hemiplegia, and episodic ataxia.^26^ EAAT2 is specifically localized in astrocytes and distributed throughout the brain and spinal cord.^25^ EAAT2 is the main glutamate transporter in the central nervous system of adult rodents. It absorbs the most extracellular glutamate (~90%) in almost all brain regions.^27^, ^28^ Studies have shown that EAAT2 keeps extracellular glutamate concentrations low and prevents neurotoxicity of glutamate, which would otherwise be elevated enough to cause epilepsy and cell death.^29^ EAAT3 protein is widely distributed in the somata and dendrites of all hippocampal neurons, and its concentration in the hippocampus of adult rats is 100 times lower than that of EAAT2.^30^ EAAT3 mainly mediates the uptake of cysteine and glutamate in the brain and plays an important role in regulating glutamatergic and GABAergic neurotransmission and neuronal redox homeostasis.^31^ Neuronal death and the number of degenerating neurons in the hippocampus more than doubled after TBI in EAAT3 (−/−) mice compared to wild‐type mice, as did superoxide production, zinc translocation, and microglial activation.^32^ EAAT4 and EAAT5 expressions are almost exclusively restricted to specific neuronal populations in the cerebellum and retina, respectively.^33^

The efficiency with which EAATs take up and transport glutamate into the intracellular astrocyte space is related to the intracellular concentration of sodium and glutamate, which may slow the cycling rate of EAATs. Glutamate absorbed into astrocytes is catalyzed by glutamine synthetase (GS) to synthesize glutamine via ammonia and ATP metabolism. Specific enzymes such as GS maintain glutamate concentrations at low levels. Its conversion to glutamate is essential to ensure the efficient clearance of glutamate by EAATs and prevent glutamate spillover.^34^, ^35^ In addition, glutamate can also enter mitochondria for oxidative metabolism. Glutamate dehydrogenase (GDH) catalyzes the initial oxidation step by deaminating glutamate to the tricarboxylic acid (TCA) cycle intermediate a‐ketoglutarate using NAD (P) 1 as a cofactor.^36^ Studies in mutant mice and allosteric drugs have shown that loss or overexpression of GDH activity in the brain modulates whole‐body energy metabolism and affects the early onset of Parkinson's disease, Alzheimer's disease, temporal lobe epilepsy, and spinocerebellar atrophy.^37^ Glutamine is released from astrocytes to extracellular space by various Na^+^ dependent and Na^+^ independent transporter pathways.^38^ The SNAT (sodium‐coupled neutral amino acid transporter) family encoded by solute carrier family 38 (SLC38) is involved in this process, with SNAT3 and SNAT5 being the primary mediators of glutamine efflux in astrocytes.^39^ Studies have shown that astrocytes adjacent to glutamatergic synapses can release glutamine in a temporally precise, controlled manner in response to the activation of glial glutamate transporters. Astrocytes can respond to synaptic activation by this mechanism and in ways that maintain or enhance further communication.^40^ The release of glutamine caused by EAATs activation is mainly driven by increased intracellular Na^+^ concentration.^41^ In addition, glutamate uptake by neurons is mediated by SNAT1 and SNAT2 on the plasma membrane of neurons,^42^ and SNAT6 has recently been shown to have a potential role in glutamate uptake at the presynaptic end of excitatory neurons.^43^ Glutamine is taken up by excitatory neurons and catalyzed by phosphate‐activated glutaminase (PAG) to produce glutamate, which continues to play its role as an excitatory neurotransmitter. It has been shown that PAG concentration and activity per neuron increase in the hippocampal formation in patients with mesial temporal lobe epilepsy (MTLE). This increase contributes to the disruption of glutamate homeostasis in the hippocampus of MTLE patients.^44^

**FIGURE 1:**
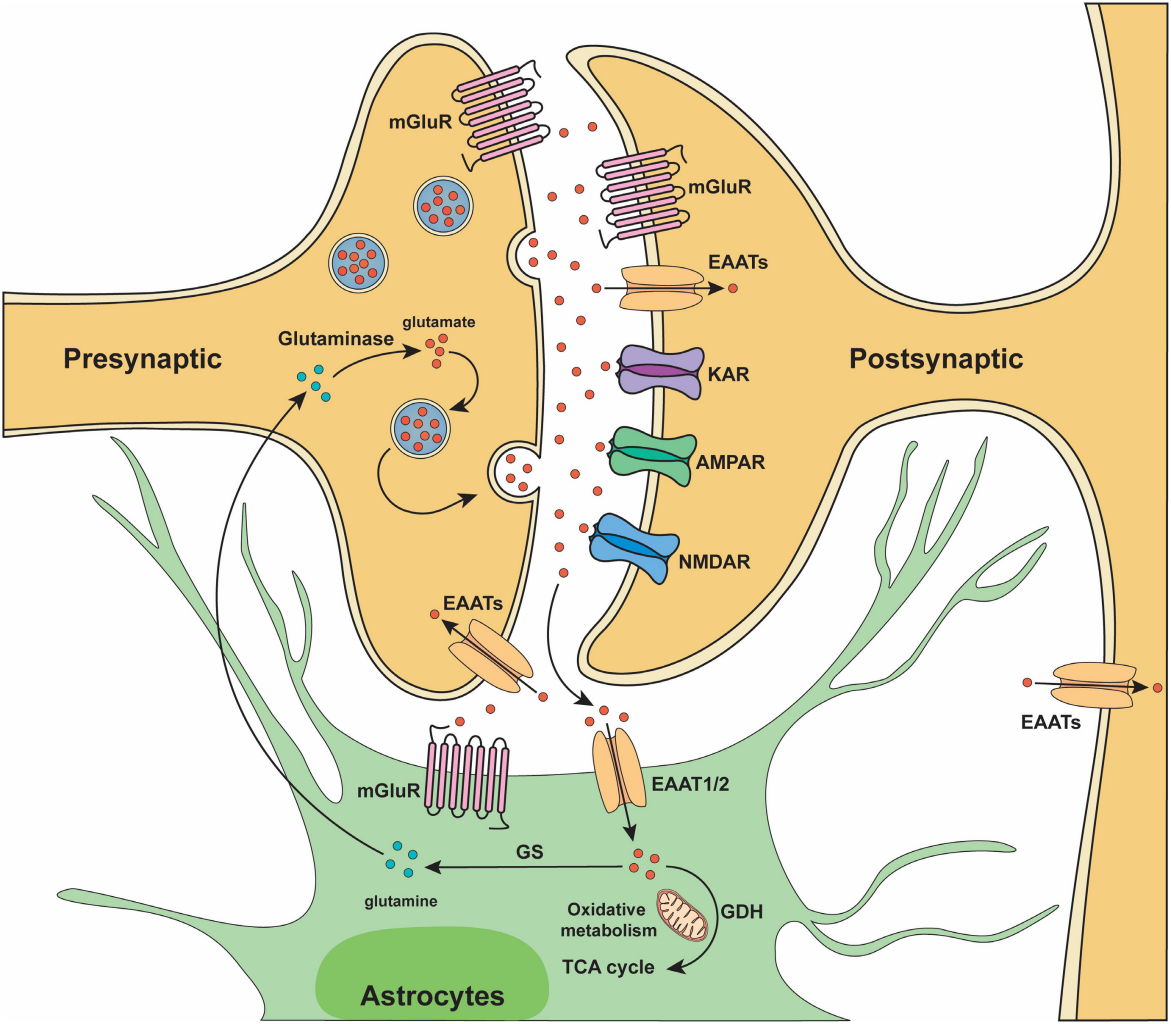
Glutamate‐glutamine cycle.

The process by which glutamate is efficiently cleared from the extracellular space and replenished at axon terminals is collectively referred to as the glutamate‐glutamine cycle and includes glutamate release, uptake, conversion, and elimination (Figure 1).^45^ Under pathological conditions, the excessive release of glutamate and the inhibition of its reuptake will cause abnormal accumulation of extracellular glutamate. High glutamate concentrations will cause excessive stimulation of ionic glutamate receptors, cause excitotoxicity, and lead to neuronal injury and death.^46^ Although NMDA receptor antagonists can logically prevent the effects of excessive activation of receptors, they have shown other side effects or little clinical benefits in clinical trials,^47^ and AMPA receptor modulators have a similar situation.^48^ Most clinical trials of NMDA antagonists have been conducted in stroke, TBI, and dementia. Still, there have been no drugs of therapeutic value, and several of the synthetic NMDA antagonist development programs have been abandoned owing to concerns about drug toxicity.^49^ Therefore, it is a practical and feasible strategy to regulate glutamate homeostasis through pharmacological methods to reduce the abnormal accumulation of glutamate caused by a pathological state and to reduce the nerve injury caused by glutamate excitotoxicity.

### 2.2 Effect of TBI on glutamate homeostasis

#### 2.2.1 Effect of TBI on glutamate release

The excessive release of glutamate and the abnormality of glutamate transporter after TBI will lead to a series of central nervous system disorders, such as Parkinson's disease^50^ and epilepsy, and some mental disorders, such as depression,^51^ cognitive impairment,^52^ anxiety, and alcohol consumption.^53^ The changes in glutamate after TBI show specific characteristics. There is a significant gender difference in glutamate production after severe TBI. Within 48 h after injury, compared with females, the production of glutamate in the cerebrospinal fluid (CSF) of males is significantly increased.^54^, ^55^ Changes in glutamate concentrations in the brain may depend on the time elapsed after injury and region‐specific.^56^ In mice, susceptibility to early and late seizures increased after repeated mild TBI. At 1 week after TBI, the increase of glutamate dominated excitation/inhibition (E/I) ratio, but 6 weeks after TBI, the glutamate levels were normalized and the imbalance of E/I ratio was characterized by the decrease of GABA.^57^ Tonic glutamate release was measured in the prefrontal cortex, dentate gyrus and striatum 2 days after mild or moderate brain injury in adult male rats using enzyme‐based microelectrode arrays. The results showed that tonic glutamate levels significantly correlated with injury severity in the dentate gyrus and striatum.^58^ TBI also affects kir4.1 and EAAT2 gene expression age‐ and time‐dependent, leading to more glutamate accumulation at early synapses in aged mice compared to adult mice, which may be related to the early and severe neuronal depolarization and excitotoxicity caused by TBI in older adults.^59^

A prospective study of 165 severe TBI patients showed that initially high glutamate values after TBI predicted poor outcomes. Compared to patients whose glutamate levels tended to normalize over the monitoring period (120 h), patients whose glutamate levels tended to increase with time or remain abnormally elevated had a higher mortality rate (39.6 vs 17.1%) and a worse 6‐month functional outcome among survivors (20.7 vs 41.2%).^60^ Glutamate chemical exchange saturation transfer (GluCEST) MRI can noninvasively image glutamate in vivo with high sensitivity and spatial resolution. Sequential GluCEST imaging scans were performed in adult male Sprague Dawley rats before TBI and 1, 3, 7, and 14 days after TBI. Compared with the baseline and control groups, GluCEST% increased and peaked on the first day after TBI in the injured cortical core lesion and peaked on the third day in the ipsilateral hippocampus. GluCEST% gradually decreased to baseline on the 14th day after TBI.^61^ GluCEST was used to evaluate glutamate changes in patients with acute mild to moderate TBI. The results showed that the level of Glutamate increased significantly during acute TBI and was closely related to the cognitive effects 1 month after injury.^62^ A prospective study was conducted on 42 severely injured adults using proton magnetic resonance spectroscopy (MRS) to measure the quantitative metabolite changes in the early (7 days) after typical brain injury and to predict the long‐term prognosis of the nervous system. The results showed that glutamate/glutamine and choline in occipital gray matter and parietal white matter were significantly increased in patients with poor long‐term (6–12 months) prognosis, which may be a reflection of early excitotoxic injury and of injury associated with membrane disruption secondary to diffuse axonal injury.^63^ The glutamate value of extracellular fluid (ECF) increased immediately after TBI, and the sustained activation of microglia was prolonged 10 days after TBI. This activation occurs after significantly increasing glutamate levels in ECF after trauma.^64^ Glutamate content increased significantly 20 min after controlled cortical impact (CCI), which coincided with the decrease of extracellular glucose (20 min) and the increase of lactic acid (40–60 min) in both brain regions after CCI.^65^ Moderate FPI (fluid percussion injury, 3.8–4.8 atm) did not change the excitability of the postsynaptic membrane of CA1 pyramidal neurons; However, glutamatergic excitatory synaptic transmission was enhanced in CA1 pyramidal neurons after FPI. Paired pulse facilitation (PPF) was significantly inhibited in the ipsilateral FPI group and contralateral FPI group. The frequency of MEPSP in neurons of the bilateral FPI group was higher than that of the control group. These results suggest that glutamatergic synaptic transmission in the hippocampal CA1 region is promoted by the presynaptic mechanism after brain injury.^66^

#### 2.2.2 Effects of TBI on glutamate transports

Morphometric immunohistochemical analysis showed that EAAT1 and EAAT2 were mainly expressed in astrocytes of the normal human neocortex. After TBI, the number of EAAT2‐positive cells in the injured neocortex and the area around the contusion decreased during a long survival period. The number of GFAP‐positive astrocytes decreased in the first 24 h. After that, the number of GFAP‐positive astrocytes increased again, indicating the formation of reactive gliosis. Double immunofluorescence showed that the absolute number of GFAP‐positive astrocytes co‐expressing EAAT1 or EAAT2 decreased when the survival time was up to 7 days. In addition, the relative proportion of astrocytes co‐expressing glutamate transporters decreased after TBI, which indicates that the decrease of EAAT1 and EAAT2 expression in posttraumatic cells is mainly due to the degeneration of astrocytes and the down‐regulation of living astrocytes. The reduction of glutamate uptake by astrocytes will aggravate the increase of extracellular glutamate after human trauma.^67^ Adult male Wistar rats received unilateral moderate FPI. The hippocampus and cortex were analyzed 3 h after the injury. It was found that the mitochondrial function was abnormal, characterized by potential membrane interruption, imbalance of the redox system, decreased mitochondrial activity, and complex I inhibition. In addition, it was also noted that the destruction of calcium homeostasis and the increase of mitochondrial swelling and mitochondrial dysfunction led to the decrease of glutamate uptake and glial glutamate transporters (EAAT1 and EAAT2), resulting in excitotoxicity.^68^ The TBI model of adult male rats was constructed by LFPI. The results showed no difference in the expression of EAAT2 in the ipsilateral cortex and hippocampus 24 h after TBI. In contrast, glutamate uptake in the cerebral cortex decreased 24 h after injury, but glutamate uptake in the hippocampus did not decrease.^69^ In addition, TBI also affects the activity of glutamate transporters. As early as 5 min after brain injury in rats with brain injury, the activity (Vmax) of EAAT2 in the cortex and hippocampus was significantly lower than that in sham‐injured animals. Still, its affinity for glutamate (Km) and the expression of glutamate transporter 1 (EAAT2) and glutamate aspartate transporter 1 (EAAT1) was not changed due to injury.^70^

Excitatory amino acid carrier type 1 (EAAC1, EAAT3) is a high‐affinity glutamate transporter that can consume energy to transfer glutamate to neurons. However, under normal physiological conditions, EAAT3 has no significant effect on glutamate clearance but participates in the cysteine uptake by neurons. This process is crucial for maintaining neuronal antioxidant function by providing cysteine to synthesize glutathione.^32^ The expression of glutamate transporter EAAT4 in the hippocampus increased 7 days after FPI. Immunohistochemical analysis showed that this increase was limited to the cells showing the morphological characteristics of astrocytes. In addition, on the third day after the injury, increased EAAT4 immunoreactivity was observed in astrocytes of the ipsilateral cortex and cerebellum, which lasted until the seventh day after the injury, indicating that this protein may play an essential role in the pathophysiology of TBI.^71^

### 2.3 Glutamate excitotoxicity and PTE

There is a close relationship between neuronal injury triggered by glutamate and excessive calcium influx. Calcium overload can trigger a series of downstream neurotoxic cascades, including the decoupling of mitochondrial electron transfer in ATP synthesis, enzyme activation, and over stimulation, such as calpain and other proteases, protein kinase, nitric oxide synthase (NOS), calcineurin and endonuclease. Changes in the activity of these enzymes will lead to (1) increased production of toxic reactive oxygen species (ROS), such as nitric oxide (NO), superoxide (O^2−^), and hydrogen peroxide, and (2) changes in cytoskeleton tissue, (3) cell death (apoptosis) and (4) activation of legacy signals of mitochondrial dysfunction.^72^ Excessive glutamate receptor stimulation can enlarge the volume of pathological tissue through the excitotoxic process.^73^ At the same time, seizures also increase the release of glutamate. Studies have shown that the extracellular glutamate concentration in rat hippocampus during pilocarpine‐induced seizures increases from ~2 to ~4 μM.^74^

The concentration of glutamate in microdialysate of 6 patients with complex partial epilepsy before and during seizures was measured by microdialysis in the bilateral hippocampus. Before seizures, the concentration of glutamate in the epileptic hippocampus was higher. During seizures, the concentration of extracellular glutamate in the epileptic hippocampus increases continuously. In addition, this increase precedes seizures, which indicates that the increase in extracellular glutamate concentration may lead to seizures.^75^ Brain microdialysis probes were implanted into 17 adults with severe brain injury, and systematic sampling was performed 1–9 days after injury. In each patient, a transient increase in glutamate was seen daily. However, these elevations were most common on day 3. These increases are often associated with seizure activity.^76^

In animal experiments, it was found that microperfusion of 200 μM glutamate into the hippocampus of free‐moving rats was sufficient to lower the picrotoxin seizure threshold down to 50% in 60% of the animals studied and prolonged seizure duration (180 ± 23%).^77^ High‐speed glutamate biosensor imaging showed that the glutamate signal transduction in the damaged cortex of TBI animals was significantly increased, and the increased glutamate response was related to epilepsy‐like activity, which was the highest when it was directly adjacent to the injury and spread through the deep cortex. The immunoreactivity of GABAergic interneuron markers decreased significantly in the whole TBI animal cortex. After CCI injury, the frequency of spontaneous inhibitory postsynaptic current decreased, and spontaneous excitatory postsynaptic current increased. It indicates that the specific microcirculation of cortical neurons may trigger and promote the spread of epileptiform activity after TBI.^78^ Disseminated depolarization within a few days after TBI can also lead to increased extracellular glutamate, increased anaerobic metabolism, and energy substrate consumption, which is related to non‐ischemic glutamate excitotoxicity and severe metabolic crisis in patients with severe TBI.^79^ An in vitro experiment confirmed that exposure to 5 μM glutamate for 30 min resulted in dead neuronal subsets and a larger number of surviving damaged neurons. Neuronal injury is characterized by persistent reversible membrane depolarization, loss of synaptic activity, and neuronal swelling. The surviving neurons in the neural network show spontaneous, repeated, and epileptic discharges, which are characterized by paroxysmal depolarization shift and high‐frequency spike–wave discharge and persist throughout the life cycle of the culture.^80^

The development of acquired epilepsy, represented by PTE, usually includes three stages: the first is the injury of the central nervous system, the second is the incubation period of epilepsy, and third is the development into spontaneous recurrent epilepsy, that is chronic epilepsy. The damage leading to acquired epilepsy often has a common molecular mechanism; that is, the increase of extracellular glutamate concentration leads to the increase of intracellular neuronal calcium, which leads to neuronal damage and/or death. Neurons that survive glutamate‐induced injury and are exposed to increased [Ca^2+^]_i,_ are cellular substrates for the development of epilepsy. Permanent long‐term plasticity changes persist in [Ca^2+^]_i_ and calcium homeostasis mechanisms of neurons surviving after injury, which has become a prominent feature of epilepsy phenotype.^5^ In vitro experiments show that TBI leads to an abnormal response of neurons to glutamate stimulation. This change in sensitivity to stimulation may be related to the changes in excitability in the first few hours to days after TBI. It may play a role in the early posttraumatic seizures in TBI patients.^81^

## 3 POTENTIAL INTERVENTION APPROACHES TO RESTORE GLUTAMATE HOMEOSTASIS AFTER TBI

Numerous studies have reported the molecular pathways that affect glutamate homeostasis after TBI (Table 1). These studies mainly focused on reducing glutamate excitotoxicity by reducing brain glutamate concentration after TBI, thereby reducing further damage and related complications caused by TBI. These molecular pathways may serve as potential drug targets to reduce the risk of PTE after TBI.

**TABLE 1:** Potential intervention pathways for restoring glutamate homeostasis after TBI and brain‐protective effects.

Intervention pathway	Mode of action	Effects on brain and glutamate homeostasis	References
NAAG peptidase	NAAG peptidase inhibition	Inhibited glutamate release and reduced acute neuropathological injury and long‐term cognitive deficits associated with TBI induced by glutamate neurotoxicity after TBI	83, 84, 85, 86, 89
Adenosine receptor	A_1A_R activation	Reduced nerve function defect, brain edema, and inflammatory cell infiltration	99
	A_2A_R and A_2B_R inhibition	Inhibited glutamate release and reduced cell injury induced by glutamate; anti‐inflammatory effect; increased the activity of Na^+^‐K^+^‐ATPase, reduced its interaction with EAAT in isolated cerebral capillaries, and restored the normal transport function of EAAT	93, 94, 95, 96, 97
Blood glutamate clearance	Intravenous injection of GOT and GPT	Mediate the redistribution of excess glutamate from brain extracellular fluid to blood, and reduce the secondary brain injury caused by glutamate neurotoxicity	103
	Administration of estrogen	Reduce the level of blood glutamate in TBI rats to improve neurological prognosis	104
	Administration of blood glutamate scavengers (oxaloacetic acid, pyruvic, GOT)	Reduced glutamate levels, improved neurologic recovery, and improved depressive‐like behavior; reduced the loss of hippocampal neurons and improved neurological function; reversed LTP inhibition by enhancing glutamate homeostasis in BBB	105, 106, 107
Regulation of metabolism	Administration of glucagon	Activated gluconeogenesis by increasing the liver uptake of amino acids such as glutamate and promoting their conversion to glucose	109
	Exogenous lactic acid supplementation	Reduced cerebral glucose consumption; Increased brain extracellular pyruvate and glucose availability and decreased brain glutamate and intracranial pressure; exerts beneficial effects on brain metabolism and hemodynamics after TBI	110
	Increased cerebral glucose concentration	Reduced brain glutamate concentration	111
Calcium influx pathway	Inhibition of calcium influx	Reduced mitochondrial injury; reduced glutamate release, and increased neuronal and astrocyte survival	116
Other molecular pathways			
Rho kinase (ROCK)	ROCK inhibition	Attenuated the decrease of EAAT2 caused by TBI; reduced the significant increase in stress‐induced glutamate levels 7 days after TBI; reduced depressive behavior induced by TBI	51
Pax3	Reduced Pax3 acetylation by restoring the interaction between HDAC4 and Pax3 in the hippocampus	Prevented the abnormal induction of vGluT1 positive glutamatergic neurons by reducing the level of Ngn2; enhanced the balance between GABAergic and glutamatergic neurons after TBI; improved anxiety, depression‐like behavior, and memory function after TBI	117
P2Y1	Adding selective P2Y1 receptor blocker to microdialysis perfusate	Reduced the levels of ATP and glutamate before the injury and eliminate the post‐CCI peak	65
Cx43	Cx43 phosphorylation inhibition	Increased expression of the glutamate transporter EAAT2 and decreased neuronal autophagy	118
p53/GLS‐2	p53 inhibition	Reduced glutamate and protein levels of glutaminase 2 (GLS‐2); protected against TBI‐induced brain injury by regulating glutamate‐mediated oxidative stress	119

### 3.1 NAAG peptidase

N‐acetyl aspartate glutamate (NAAG) is a ubiquitous neuropeptide in the central nervous system. It is released with glutamate and inhibits presynaptic glutamate release by acting on mGluR3. Extracellular NAAG is hydrolyzed to N‐acetyl aspartic acid and glutamate by peptidase [glutamate carboxypeptidase II (GCP II) and GCP III] activity.^82^ Studies have shown that NAAG peptidase inhibitor ZJ‐43 can increase the extracellular NAAG level and reduce the extracellular levels of amino acid neurotransmitters, including glutamate, aspartic acid, and GABA in the dialysate of the dorsal hippocampus of TBI through the mechanism mediated by group II metabotropic glutamate receptor (mGluR).^83^ Systemic injection of GCP II inhibitor ZJ‐43 and its prodrug PGI‐02776 within the first 30 min after the injury can reduce the acute neuropathological and TBI‐related long‐term cognitive impairment caused by excessive extracellular glutamate,^84^ significantly reduce the harmful effect of TBI combined with secondary hypoxic injury,^85^ and reduce the exercise and cognitive impairment combined with TBI and the second hypoxic injury within a few weeks after injury.^86^

GCP II is a transmembrane zinc metallopeptidase that mainly exists in the nervous system, prostate, and small intestine. In the nervous system, glial cell‐bound GCP II mediates the neurotransmitter NAAG hydrolysis to glutamate and N‐acetyl aspartate. Studies have shown that inhibition of GCP II can attenuate excitotoxicity associated with enhanced glutamate transmission under pathological conditions. The sensitivity of GCP II‐KO mice to moderate TBI was significantly reduced.^87^ Two non‐invasive methods, 1H‐MRS and T2 MR imaging, as well as in vitro brain water content measurement, showed that the reduction of glutamate and N‐acetyl aspartate levels were less obvious in GCP II‐KO mice than in WT mice, proving that the potential mechanism of the neuroprotective effect of GCP II‐KO on TBI brain swelling involves the changes of glutamate and N‐acetyl aspartate levels.^88^ In the animal model of TBI, inhibition of GCP II can increase the extracellular level of NAAG, inhibit the release of glutamate, and have neuroprotective effects. This effect was confirmed in GCP II‐KO mice by reducing oxidative stress.^89^

### 3.2 Adenosine receptor

After the severe craniocerebral injury, the concentration of adenosine in cerebrospinal fluid increased in a time‐dependent and severity‐dependent manner. There is a strong correlation between increased CSF adenosine and glutamate concentrations, which may reflect endogenous neuroprotective measures against excitatory toxicity after severe TBI.^90^ Evidence suggests that early release of adenosine after TBI can inhibit seizures and brain inflammation, while glutamate can increase intracellular and extracellular adenosine release.^91^ Adenosine plays a dual role in glutamate cytotoxicity, in which A_2A_ adenosine receptor (A_2A_R) and A_2B_ adenosine receptor (A_2B_R) activation intensify glutamine‐induced cell injury, while A_1_ adenosine receptor activation ameliorates glutamine‐induced cell injury.^92^ Studies have shown that the glutamate level in the CSF of A_2A_R‐KO mice was significantly lower than that of WT littermates, and A_2A_R gene knockout showed a neuroprotective role in the TBI mouse model by inhibiting glutamate release.^93^, ^94^ The protective effects of selective inactivation of A_2A_R in bone marrow‐derived cells (BMDCs) or widespread inactivation of A_2A_R in non‐BMDCs are accompanied by decreased CSF glutamate levels and inhibition of inflammatory cytokines IL‐1 or IL‐1 and TNF‐α, thereby exerting a protective effect on TBI.^95^ The switch in the effect of A_2A_R activation from anti‐inflammatory to pro‐inflammatory depends on glutamate concentration.^96^ After TBI, inhibition of adenosine receptors increases Na^+^‐K^+^‐ATPase activity and reduces its interaction with EAATs in isolated brain capillaries, restoring the normal transport function of EAATs.^97^ In addition, the high level of blood glutamate after TBI is also closely related to the occurrence and severity of traumatic brain injury‐induced acute lung injury. The combination of an A_2A_R activator and exogenous glutamate can aggravate lung injury, indicating the correlation between A_2A_R activation and glutamate excitotoxicity.^98^

Caffeine is the most consumed psychoactive drug and non‐specific adenosine receptor antagonist. In the CCI‐induced TBI mouse model, long‐term (3 weeks) caffeine preconditioning in drinking water significantly reduced neurological deficits, brain edema, and inflammatory cell infiltration compared with the control group. Its mechanism of action may be achieved by upregulating brain A_1_ receptor mRNA and inhibiting excessive glutamate and inflammatory cytokine release.^99^

### 3.3 Blood glutamate clearance

Studies have shown that excessive glutamate in the brain can be eliminated by reducing the level of glutamate in the blood and accelerating the outflow of brain–blood glutamate.^100^ After intravenous injection of non‐metabolic analogs of glutamate to juvenile rats, it can be observed that glutamate is rapidly cleared from the blood to peripheral tissues in a non‐metabolic form. Among them, the liver plays a central role in glutamate metabolism. It is the source of glutamate metabolites, which will be redistributed to skeletal muscle and intestine, which suggests that drug manipulation reduces the release rate of glutamate in the liver or increases the pumping rate of glutamate in skeletal muscle, may achieve neuroprotective effects in the brain.^101^ Some studies have shown that the transient decrease of blood glutamate after TBI is a stress response, and β2 is a result of the activation of the sympathetic nervous system by adrenergic receptors. The observed increase in glutamate brain blood outflow leads to a high level of glutamate in the brain after brain injury.^102^ Intravenous injection of glutamic oxaloacetic transaminase (GOT) and glutamic pyruvate transaminase (GPT) can reduce the level of blood glutamate. The lowering effect of GOT and GPT on glutamate can mediate the redistribution of excess glutamate from brain extracellular fluid to blood and reduce the secondary brain injury caused by glutamate neurotoxicity.^103^ Injection of Premarin into male rats can significantly reduce the blood glutamate level in TBI rats. This decrease is related to the improvement of neurological prognosis, suggesting the role of estrogen in neuroprotection.^104^ Administration of pyruvate to clear blood glutamate after TBI has been widely proven to be an effective method to provide neuroprotection by reducing blood glutamate and subsequent brain glutamate levels. Studies have shown that prophylactic or therapeutic administration of pyruvate after TBI can reduce glutamate levels and improve the recovery of the nervous system and TBI‐induced depression‐like behavior.^105^ Using blood glutamate scavengers, oxaloacetic acid, and pyruvic acid can also provide a neuroprotective effect after TBI, which reduces the loss of hippocampal neurons and improves neurological function.^106^ Delayed cognitive impairment after brain injury is more related to transient glutamate enhancement in the hippocampus. The peripheral glutamate scavenger rGOT can reverse LTP inhibition by enhancing glutamate homeostasis in BBB.^107^

### 3.4 Regulation of metabolism

The endogenous mechanism of decreasing glutamate concentration in the brain parenchyma after brain injury is unclear. Mass spectrometric evidence shows that glutamate oxidation can reduce glutamate concentration through a “truncated” tricarboxylic acid cycle coupled with the urea cycle. This process reduces glutamate levels, generates carbon for energy metabolism, leads to citrulline accumulation, and produces nitric oxide.^108^ Glucagon can activate gluconeogenesis by increasing the liver uptake of amino acids such as glutamate and promoting their conversion to glucose. Studies have shown that glucagon plays a prominent neuroprotective role after TBI by reducing glutamate in the central nervous system. Despite elevated blood glucose, glucagon is also beneficial.^109^ Exogenous lactic acid supplementation can be used as the priority energy substrate of the injured human brain to save brain glucose consumption. It can also increase the availability of brain extracellular pyruvate and glucose and reduce brain glutamate and intracranial pressure, indicating that hypertonic lactate treatment has beneficial brain metabolism and hemodynamics after TBI.^110^ In addition, there is experimental evidence that brain glutamate was lowest measured at brain glucose 3–5 mM and increased significantly at brain glucose below 3 mM or above 6 mM. Insulin administration could increase brain glutamate at low brain glucose.^111^ However, reducing excitatory amino acids after ischemia by adding glucose to dialysate had no significant effect on dialysate glutamate in the cortex or hippocampus after TBI.^112^

### 3.5 Calcium influx pathway

Calcium can mediate the release of glutamate and the interruption of glial regulation of extracellular glutamate, resulting in the increase of extracellular glutamate in the striatum 2 days after diffuse brain injury.^113^ TBI‐induced glutamate release increases mitochondrial Ca^2+^ cycle/overload, resulting in mitochondrial dysfunction.^114^ Mitochondrial dysfunction will further reduce glutamate uptake and glial glutamate transporters (EAAT1 and EAAT2), leading to excitotoxicity. Guanosine (GUO) treatment can improve mitochondrial damage and glutamatergic dysfunction as an effective strategy secondary to acute physiology after TBI.^68^, ^115^ In addition, the acute increase of intracellular [Ca^2+^]_i_ after TBI can also trigger the cellular mechanism leading to neuronal dysfunction and death. Administration of the N‐type voltage‐gated calcium channel (VGCCs) blocker SNX‐185 before or immediately after cell injury reduced glutamate release and increased neuronal and astrocyte survival. Delayed treatment did not improve cell survival but significantly promoted [Ca^2+^]_i_ return to baseline.^116^

### 3.6 Other molecular pathways

TBI allows thrombin extravasation. Applying the thrombin receptor PAR‐1 agonist could cause the down‐regulation of EAAT2 while inhibiting Rho kinase (ROCK) prevented this process. Evidence showed that the activity of thrombin in the hippocampus of mice increased 1 day after closed skull TBI. Administration of PAR‐1 antagonist SCH79797 could prevent the decrease of EAAT2 and EAAT1 expression in the hippocampus 7 days after TBI. The inhibition of rock attenuated the decrease of EAAT2 after TBI but did not attenuate the decrease of EAAT1. The significant increase in stress‐induced glutamate levels 7 days after TBI was reduced by applying rock inhibitor fasudil. At the same time, inhibition of PAR‐1 or rock reduced the induced depressive behavior after TBI.^51^

TBI mediates the activation of transcription factor Pax3 by increasing its acetylation state and subsequently induces Ngn2 transcription. In turn, this event enhanced the vGlut1‐expressing glutamatergic neurons and the accumulation of excess glutamate in the hippocampus. The results showed that the acetylation of Pax3 increased due to the loss of interaction with the down‐regulated histone deacetylase 4 (HDAC4) after TBI. TBI‐induced GSK3β activation is the cause of HDAC4 degradation. Overexpression of HDAC4 in TBI can reduce Pax3 acetylation by restoring the interaction between HDAC4 and Pax3 in the hippocampus. This event prevented the abnormal induction of vGluT1 positive glutamatergic neurons by reducing the level of Ngn2 and then strengthened the balance between GABAergic and glutamatergic neurons after TBI, thereby improving the anxiety, depression‐like behavior, and memory function after TBI.^117^

Glutamate increased significantly 20 min after CCI, concomitant with a decrease in extracellular glucose and an increase in lactate in both brain regions after CCI. Adding a selective P2Y1 receptor blocker to microdialysis perfusate can significantly reduce the levels of ATP and glutamate before the injury and eliminate the post‐CCI peak.^65^ Connexin 43 (Cx43) is one of the major gap junction proteins in astrocytes. Phosphorylation of Cx43 (p‐Cx43) by astrocytes can regulate the level of neuronal autophagy in the hippocampus of rats after TBI. Studies have shown that Cx43 may induce neuronal autophagy and reduce the expression of EAAT2 in the hippocampus by activating the P2X7 receptor and promoting the repair of TBI‐induced cognitive defects as a therapeutic strategy to intervene TBI.^118^

Hydrogen sulfide (H2S) can protect against TBI‐induced brain injury by reducing the protein levels of glutamate and glutaminase 2 (GLS‐2) in a p53/GLS‐2 pathway‐dependent manner and regulating glutamate‐mediated oxidative stress.^119^

### 3.7 Genetic variation

Several studies have also evaluated the impact of gene polymorphism on the occurrence of epilepsy in TBI patients and have shown a particular correlation. In regulating glutamate homeostasis, the variation of genes that regulate glutamate transporters and key enzymes is a potential factor leading to PTE.

Genetic variations in neuronal glutamate transporter genes are associated with accelerated epilepsy and increased risk of PTS after severe TBI (sTBI). Two studies evaluated the occurrence of epilepsy after TBI in 253 and 267 adult patients aged 18–75 based on single nucleotide polymorphisms (SNPs), respectively. The results showed that variations within *SLC1A1* (EAAT3) and *SLC1A3* (EAAT1) increase the risk of epilepsy after sTBI, which may serve as potential drug targets for PTS.^120^, ^121^

Glutamate is converted into GABA through two types of glutamate decarboxylase, GAD67 and GAD65, encoded by the *GAD1* and *GAD2* genes, respectively. In the *GAD1* gene, the tagging SNP (tSNP) rs3828275 was associated with an increased risk of PTS within 1 week of TBI. In comparison, tSNP rs769391 and functional SNP rs3791878 in the *GAD1* gene were associated with an increased risk of PTS from 1 week to 6 months post‐TBI. Both risk variants conferred increased susceptibility to PTS compared to subjects with 0–1 risk variants.^122^ Therefore, the discovery and early identification of relevant genetic biomarkers are of great significance for preventing and treating PTE.

## 4 PHARMACOLOGICAL STUDY ON PREVENTING PTE BY RESTORING GLUTAMATE HOMEOSTASIS

A series of preclinical studies have explored the preventive and protective effects of drugs on the occurrence and development of epilepsy after TBI based on the mechanism of regulating glutamate homeostasis (Table 2).

**TABLE 2:** Pharmacological study on preventing PTE by restoring glutamate homeostasis.

Compounds	Chemical structure	Animal model	Targets/mechanism of action	Outcome/effects	References
Ceftriaxone	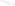	Long‐Evans rats (367 ± 36 g, age 8–9 weeks)/lateral fluid percussion injury	Reverses loss of EAAT2 after TBI and reduces increased GFAP expression.	Reduced cumulative PTS duration at 12 weeks post‐injury	126
Levetiracetam	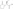	Male Wistar rats (250–280 g)/unilateral amygdala FeCl_3_ Injection	Suppresses glutamate overflow; Reduces glutamate‐induced excitotoxicity, and enhances GABAergic inhibition; increases the expression of glutamate and GABA transporters (EAAT1, EAAT2, EAAT3, GAT‐1, and GAT‐3), and reduces the expression of glutamate transporter regulatory protein GTRAP3‐18	No epilepsy occurred in the drug treatment group	127
Dehydro‐epiandrosterone (DHEA)	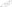	Male Wistar rats (400–450 g, age 8–10 months)/somatosensory region of the cortex FeCl_3_ injection	Reduces glutamate levels in hippocampal synaptosomes; upregulates the mRNA of glutamate transporters EAAT1, EAAT2, and EAAT3	Significantly reduced the multiple unit activity potentials (MUA) count and epileptic activity of EEG in model animals	128
Purmorphamine	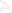	Adult wild‐type albinob4 zebrafish (both sexes, age 6–18 months, 3–5 cm in length)/modified Marmarou weight drop protocol	Sonic hedgehog (Shh) inhibitor Induces EAAT2 expression	Reduced PTS, edema, and cognitive impairment in TBI fish	129
Creatine	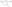	Male Wistar rats (age 90 days)/fluid percussion injury + a sub‐convulsant dose of PTZ	Against the reduction of GAD67 levels, GAD activity and specific [3H] flunitrazepam binding in the Hippocampus; reduces cell loss, including the number of parvalbumin‐positive (PARV+) in the hippocampal CA3 region	Significantly increased the latency of the first myoclonic and tonic–clonic seizure, and reduced the time of tonic–clonic seizures epileptiform discharges, and spindle oscillations caused by PTZ (35 mg/kg)	130
Phenyl‐butyl‐nitrone (PBN)	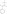	Adult male Sprague–Dawley rats (335–410 g, age 3 weeks)/intracortical FeCl_2_ injection	Radical scavenger Attenuates the decrease of 70 kDa EAAT2 and increases the arginine/citrulline ratios in iron‐induced PTE animals	Improved the redox situation in iron‐induced epileptogenesis	131

It has long been known that β‐lactam antibiotics are an effective stimulator of EAAT2 expression, which may be mediated by increasing the transcription of the EAAT2 gene. Ceftriaxone can increase the expression and biochemical and functional activity of EAAT2 in animal brains and play a neuroprotective role in animal disease models based on glutamate toxicity, such as ischemic brain injury, motor neuron degeneration, amyotrophic lateral sclerosis (ALS).^123^ Based on this mechanism, the role of ceftriaxone in post‐TBI has also been further confirmed.^124^, ^125^ Subsequent experiments showed that compared with the control group, intraperitoneal injection of ceftriaxone 200 mg/kg per day could reverse the loss of EAAT2 expression in LFPI model rats after 7 days of TBI and reduce the expression level of GFAP in the injured cortex by 43%. At 12 weeks after injury, ceftriaxone treatment reduced the cumulative seizure duration of PTE in TBI rats, indicating that ceftriaxone has a potential role in treating epileptogenic TBI.^126^

Levetiracetam (Lev) inhibited glutamate excitation and enhanced GABA inhibition in the rat epilepsy model induced by FeCl_3_ amygdala injection. Compared with epileptic rats treated with vehicle, the levels of EAAT3 and GAT‐3 in epileptic rats treated with Lev increased. In contrast, the levels of glutamate transporter regulatory protein (GTRAP3‐18) decreased, suggesting that Lev's antiepileptic effect may be partly due to the reduction of glutamate‐induced excitotoxicity and the enhancement of GABAergic inhibition. These conclusions are supported by the increased expression of glial glutamate transporters (EAAT1 and EAAT2) and the increased expression of EAAT3 and GAT‐3 associated with decreasing GTRAP3‐18.^127^

Dehydroepiandrosterone (DHEA) is a neuroactive androgen steroid with an antiepileptic effect in the iron‐induced PTE rat model. DHEA was intraperitoneally administered to iron‐induced epileptic rats for 7, 14, and 21 days. The results showed that the level of glutamate transporter mRNA decreased significantly during epilepsy. DHEA treatment resulted in a significant increase of EAAT2, EAAT1, and EAAT3 mRNA of glutamate transporters, indicating that DHEA treatment can induce the up‐regulation of these transporters, thus inhibiting the occurrence and development of epilepsy after brain injury.^128^

Hentig j et al. used zebrafish as the research object to explore the inhibitory effect of activating the sonic hedgehog (Shh) signal pathway on PTE induced by the modified Marmarou weight drop method and the expression of EAAT2. Studies have shown that blocking glutamate receptors or increasing the expression of glutamate transporter EAAT2a can reduce TBI‐induced PTS. Compared with an untreated control group, Smoothened agonist purmorphamine increased EAAT2a expression in the intact brain, and purmorphamine treatment reduced glutamate excitotoxicity after TBI. Similarly, purmorphamine reduced PTS, edema, and cognitive deficits in TBI fish, while cyclopamine‐treated TBI fish increased and/or prolonged these pathologies. However, co‐treatment of fish with ceftriaxone induced EAAT2a expression, thereby reducing the severity of the increased TBI phenotype of cyclopamine. These data suggest that the Shh signal induces EAAT2a expression and regulates TBI‐induced glutamate excitotoxicity and TBI sequelae.^129^

The experiment showed that creatine supplementation for 4 weeks, starting from 1 week after FPI, could significantly increase the latency of the first myoclonic and tonic–clonic seizures in model rats and reduce the duration, intensity, epileptiform discharge and spindle oscillation induced by sub convulsive dose PTZ. A creatine supplement can inhibit the level of glutamate decarboxylase GAD67 and decrease GAD activity and the specific [3H] flunitrazepam binding in the hippocampus. It suggests chronic creatine supplements may play a neuroprotective role in brain excitability by controlling GABAergic function after TBI.^130^

Samuelsson et al. found that the level of EAAT2 (70 kDa) at the lesion of iron‐induced PTE animals significantly decreased, with higher basal glutamate and lower glutamine levels as low arginine/citrulline ratios compared to controls. Phenyl‐butyl‐nitrone (PBN), a nitrone radical scavenger, attenuated the decrease of 70 kDa EAAT2 and increased the arginine/citrulline ratios in PTE animals, indicating that the administration of free radical scavengers may weaken the oxidative damage to glutamate uptake by astrocytes in iron‐induced epilepsy.^131^

## 5 CONCLUSION AND FUTURE PERSPECTIVE

This article reviews the role of glutamate homeostasis in PTE and the preventive effect of glutamate homeostasis regulation on PTE after TBI. There is a latent period between brain injury and PTE, a series of molecular biological changes caused by tissue injury, which provides a window for drug intervention in preventing late epilepsy. The mechanism of PTE is complex, involving multiple causes such as neuroinflammation, oxidative stress, and excitotoxicity. Excitotoxicity caused by glutamate is a vital link that causes neuronal death and neural network remodeling. The drugs listed in this article may not play a role in PTE prevention simply by regulating glutamate homeostasis. Some drugs have other neuroprotective effects at the same time. The role of regulating glutamate homeostasis in various animal models of epilepsy and TBI has been confirmed, but the role of regulating glutamate homeostasis in the prevention of PTE is still less studied. In addition, in pharmacological research, PTE often uses the same or similar animal models as TBI, such as FPI and CCI, which also suggests that some drugs that take effect in TBI experiments may also play a particular role in the prevention and treatment of PTE, which is worthy of further pharmacodynamic research. Clarifying the relationship between TBI and PTE can also provide a theoretical basis for the application of drugs to prevent TBI in the prevention of PTE. Finally, possible molecular targets or biological pathways to restore glutamate homeostasis after TBI were reviewed. It is known that various nervous system diseases, such as PTE, Parkinson's disease, depression, and cognitive impairment, are related to the abnormal level of glutamate. Taking this as a research target to develop new drugs may play a neuroprotective role while avoiding the side effects caused by the direct inhibition of glutamate receptors.

## FUNDING INFORMATION

This work was supported by the Ningxia Medical University Scientific Research Project (XZ2021001), the Key Research and Development General Project of Ningxia Hui Autonomous Region (2018BEG03013), and the Open Project of Ningxia Key Laboratory of Craniocerebral Diseases of Ningxia Medical University (LNKF202201).

## CONFLICT OF INTEREST STATEMENT

The authors declare that they have no competing interests.

## Data Availability

Data sharing is not applicable to this article as no new data were created or analyzed in this study.
